# Radio(chemo)therapy in anal cancer: evaluation of sex-specific disparities across AJCC stages

**DOI:** 10.1007/s00066-025-02368-1

**Published:** 2025-02-07

**Authors:** F. Fuchs, P. Rogowski, M. Rottler, M. A. Shouman, K. Heinrich, F. Kühn, C. Belka, K. Unger, F. Walter

**Affiliations:** 1 Department of Radiation Oncology, LMU University Hospital, LMU Munich, Munich, Germany; 2 Department of Internal Medicine III, LMU University Hospital, LMU Munich, Munich, Germany; 3 Partner Site Munich, German Cancer Consortium (DKTK), Munich, Germany; 4 Department of General, Visceral, and Transplantation Surgery, LMU University Hospital, LMU Munich, Munich, Germany; 5 Bavarian Cancer Research Center (BZKF), Munich, Germany

**Keywords:** Anal carcinoma, Chemoradiotherapy, Sex disparities, Gender medicine, AJCC staging system

## Abstract

**Purpose:**

This study aimed to investigate sex differences in anal squamous cell carcinomas (ASCC), with a particular focus on the prognostic significance of the American Joint Committee on Cancer (AJCC) 9th edition staging system for oncological outcome.

**Methods:**

A retrospective analysis was conducted in 188 patients with histologically confirmed ASCC who underwent definitive (chemo)radiotherapy between 2004 and 2020. Patient- and tumor-related data were collected. Tumor stage groups were classified according to the AJCC 9th edition. Overall survival (OS), disease-free survival (DFS), freedom from recurrence (FFR), and colostomy-free survival (CFS) were analyzed using the Kaplan–Meier method for univariate testing and Cox regression models for multivariate analysis. Differences between sexes were assessed.

**Results:**

The cohort included 134 females and 54 males, with a median follow-up of 83 months. Females exhibited significantly better OS (*p* = 0.01), DFS (*p* = 0.01), and CFS (*p* = 0.03). For male patients, there was a clear trend towards better OS (*p* = 0.08), DFS (*p* = 0.10), and FFR (*p* = 0.09) in earlier tumors as well as significantly better CFS (*p* = 0.04). In contrast, in the female subgroup, there were no significant differences in OS (*p* = 0.64), DFS (*p* = 0.52), and CFS (*p* = 0.25) with respect to tumor stage. In multivariate analysis, male sex, older age, and advanced tumor stages were significant risk factors for poorer OS, DFS, and CFS.

**Conclusion:**

This study highlights significant sex differences in ASCC prognoses, with females showing better survival outcomes. The prognostic value of the AJCC 9th edition staging system differs between sexes; thus, we support the inclusion of sex as a prognostic factor in staging systems.

**Supplementary Information:**

The online version of this article (10.1007/s00066-025-02368-1) contains supplementary material, which is available to authorized users.

## Introduction

Anal squamous cell carcinoma (ASCC) represents a relatively rare tumor entity, accounting for approximately 0.5% of all cancer cases. Unlike many other malignancies, such as colorectal cancer, there has been a continuous increase in both the incidence and mortality rates of ASCC over the past decades [1]. With an age-standardized incidence rate of 1.3 per 100,000 inhabitants for males and 2.3 per 100,000 for females in Germany in 2020, approximately two thirds of patients are female [2–4].

Small tumors of the anal margin can be cured with surgical resection only. However, tumors located in the anal canal, locally advanced perianal tumors, and tumors with nodal metastatic disease are usually treated with definitive (chemo)radiotherapy [5–7]. Concurrent chemoradiotherapy with 5-fluorouracil (5‑FU) and mitomycin C (MMC) is superior to radiotherapy alone in terms of local recurrence rate and overall survival for all patients, including those with T1N0 cancer of the anal canal, and is therefore the standard treatment today [6, 8, 9]. For tumor staging, the TNM staging system is applied, which underwent considerable changes with the last update in 2017 with respect to nodal involvement [10]. Most recently, the AJCC staging system was revised, and a 9th version was introduced following evidence indicating a lack of hierarchical prognostic order in the 8th version [11]. In the 9th version, stages IIB, IIIA, and IIIC were revised, suggesting that the T category has a greater effect on survival than the N category. As stated by O’Sullivan et al., the tumor staging system serves three main purposes: firstly, to determine the extent of the tumor at diagnosis, which is crucial for choosing appropriate treatment options; secondly, it acts as a prognostic tool for predicting oncologic outcomes; and thirdly, it allows for comparison of different patient groups in the context of medical research [12].

Anal squamous cell carcinoma exhibits distinct sex disparities in terms of incidence and prognosis. Approximately two thirds of patients with anal carcinoma are female, with a better prognosis than male patients, irrespective of tumor stage [13–18]. In recent years, oncology research has increasingly focused on sex and gender-specific differences. For example, differences attributable to sex have been observed in terms of the incidence, response to treatment, and survival rates of various cancers, including melanoma, colorectal cancer, and lung cancer [2, 19–22]. The underlying reasons for these disparities may include variations in tumor biology, hormonal influences, and differences in health-seeking behavior and access to healthcare services between sexes. To date, the underlying mechanisms are not fully understood, especially for rare tumor diseases such as ASCC. To gain a better understanding of these mechanisms and to ensure optimal individual treatment, sex should be considered as a significant factor in cancer research, prevention, and treatment strategies.

The aim of this study was therefore to further investigate sex differences in ASCC, with a particular focus on the prognostic significance of the AJCC 9th edition staging system on oncological outcome.

## Methods

Patients with histologically confirmed anal carcinoma undergoing definitive radiotherapy with or without concurrent chemotherapy in our clinic between 2004 and 2020 were retrospectively analyzed.

The retrospective analysis was performed in compliance with the principles of the Declaration of Helsinki and its subsequent amendments and was approved by the local Ethics Committee of the University of Munich (approval 23-0166). The requirement to obtain informed consent was waived.

Patients with metastatic disease, current palliative treatment, or prior pelvic radiotherapy were excluded. Patient- (age, sex, height, weight, HIV status, and Karnofsky performance status) and tumor-related data (TNM status, date of initial diagnosis, HPV status, etc.) were collected from patient records.

All cases were carefully reviewed and the TNM stage was determined according to the 8th edition. Subsequently, stage groups were classified according to the 9th edition of the AJCC Cancer Staging Manual. Staging was categorized into early (stage I), intermediate (stage IIA and IIB), and advanced (stage IIIA, IIIB and IIIC) stages.

Radiation parameters were extracted from the radiation plans. Follow-up was conducted using data obtained during regular radiotherapy follow-up and through telephone patient interviews.

### Treatment

All patients received definitive radiotherapy with or without concurrent chemotherapy. Due to the extended time period covered by this study, target delineation, radiation technique, and dose prescription varied to some extent. Prior to radiotherapy, patients underwent staging via digital rectal examination, proctoscopy including biopsy, and CT of the thorax and abdomen. In the majority of patients, pelvic MRI and/or FDG positron-emission tomography (PET/CT) were also performed. All patients were treated with three-dimensional conformal radiotherapy (3D-CRT), and starting from 2010, IMRT/VMAT was introduced. Either a sequential or a simultaneously integrated boost was delivered to the primary and the affected lymph nodes.

The majority of patients received chemotherapy according to the regimen proposed by Flam et al., which consisted of MMC at a dose of 10 mg/m^2^ on day 1 and day 29, along with 5-FU at a dose of 1000 mg/m^2^ administered as a continuous infusion on days 1–4 and 29–32. Alternatively, some patients received capecitabine twice daily instead of 5‑FU in addition to MMC. In cases of advanced age, poor general health, and/or significant comorbidities, some patients either skipped chemotherapy or had their treatment de-escalated to MMC monotherapy.

### Statistics

Overall survival (OS) was defined as the time interval from the last day of radiotherapy to any form of death. Disease-free survival (DFS) was measured from the last day of radiotherapy to the date of local recurrence, regional recurrence, occurrence of distant metastases, or death. Freedom from recurrence (FFR) was calculated using the date of diagnosis of local or regional recurrence or the occurrence of distant metastases. Colostomy-free survival (CFS) was defined as the time interval between the last day of radiotherapy and colostomy or death. Patients who did not experience the event of interest were censored at their last follow-up date. For AJCC stage (9th edition), we used the main staging criteria AJCC stage I, AJCC stage II, and AJCC stage III. Patient age was categorized as “older” for patients aged 66 years and older and as “younger” for patients younger than 66 years.

The statistical analysis was conducted using the R statistical programming framework (version 4.3.3) within the RStudio Integrated Development Environment (IDE; R Foundation, Vienna, Austria). R packages arsenal, survminer, survival, and tableone were used.

Univariate testing assessing the impact of specified clinical and demographic factors (age, sex, T stage, N stage, AJCC stage, categorized AJCC stage, MRI) on OS, DFS, FFR, and CFS was conducted using the Kaplan–Meier method, and significance was assessed using the log-rank test. Parameters with a *p*-value less than 0.1 in univariate analysis were included in a Cox regression model for multivariate analysis. A *p*-value of < 0.05 was defined as statistically significant.

Descriptive statistics and subgroup comparisons were conducted and outputted in table format using the tableby function. For testing differences in frequency, the chi-square test was used, while the Mann–Whitney U test was employed to analyze mean differences between groups. Survival curves were visualized using the Kaplan–Meier method using the ggsurvplot function. Testing for differences in survival was conducted using Cox proportional hazards models in a univariate fashion if only one variable was considered or in multivariate fashion if more than one variable was used. For every model, the log-rank test *p*-value was reported along with the concordance index (C-index); in the case of multivariate Cox proportional hazard models, also the Wald test *p*-values were reported. Forest plots, generated using the forestploter R package, were used to visualize hazard ratios (HRs) and their 95% confidence intervals (CIs). When a stratum had no events, the CI could not be calculated and was reported as “not available” (NA). In the multivariate analysis, we included variables that showed a trend of association (*p*-value < 0.2) with at least one endpoint. Following standard biomedical practice, we considered *p*-values below 0.05 to be statistically significant.

## Results

### Patient characteristics

A total of 188 patients with a median age of 64 years (range 34–94 years) were included in this study. Patients were categorized as older (> 65 years, *n* = 86 [46%]) and younger (≤ 65 years, *n* = 102 [54%]). Overall, 29% (*n* = 54) of patients were male and 71% (*n* = 134) were female. 30 patients (16%) had AJCC stage I, 63 patients (34%) stage IIA, 32 patients (17%) stage IIB, 48 patients (26%) stage IIIA, 12 patients (6%) stage IIIB, and 3 patients (2%) stage IIIC.

While 38% (*n* = 69) had high-grade tumors, 62% (*n* = 113) had low-grade tumors. Information on grading was missing in 6 cases. HPV was not tested for in 139 cases, and of the tested cases, 6% (*n* = 3) were negative and 94% (*n* = 46) were positive. Overall, 11 patients were HIV positive, and 177 patients were either negative for HIV or information on HIV status was not available. Routine testing for HIV infection was conducted. However, since this involved genetic testing, the results were retrospectively unavailable within the clinical information system for a significant number of patients. The median follow-up period was 83 months (range 1–216 months).

An overview on the patient characteristics separated by sex is given in Table 1.

**Table 1:** Patient and tumor characteristics separated by sex

	Missing	Overall	Male	Female	*p*-value
*N (%)*	–	188	54 (28.7)	134 (71.3)	–
*Age at diagnosis, median [Q1, Q3]*	0	64.0 [54.6, 72.0]	60.8 [52.0, 69.8]	64.8 [54.8, 72.8]	0.200
*Age, n (%)*					
Older (> 65 years)	0	86 (45.7)	22 (40.7)	64 (47.8)	0.476
Younger (≤ 65 years)	–	102 (54.3)	32 (59.3)	70 (52.2)	
*T stage, n (%)*					
T1	0	34 (18.1)	5 (9.3)	29 (21.6)	0.133
T2	–	91 (48.4)	32 (59.3)	59 (44.0)	
T3	–	48 (25.5)	12 (22.2)	36 (26.9)	
T4	–	15 (8.0)	5 (9.3)	10 (7.5)	
*N stage, n (%)*					
N0	0	121 (64.4)	30 (55.6)	91 (67.9)	0.167
N1a	–	57 (30.3)	19 (35.2)	38 (28.4)	
N1b	–	9 (4.8)	5 (9.3)	4 (3.0)	
N1c	–	1 (0.5)	–	1 (0.7)	
*AJCC stage 9th edition, n (%)*					
I	0	30 (16.0)	4 (7.4)	26 (19.4)	0.176
IIA	–	63 (33.5)	22 (40.7)	41 (30.6)	
IIB	–	32 (17.0)	11 (20.4)	21 (15.7)	
IIIA	–	48 (25.5)	12 (22.2)	36 (26.9)	
IIIB	–	12 (6.4)	5 (9.3)	7 (5.2)	
IIIC	–	3 (1.6)	–	3 (2.2)	
*AJCC stage categorized, n (%)*					
AJCC stage III	0	63 (33.5)	17 (31.5)	46 (34.3)	0.073
AJCC stage II	–	95 (50.5)	33 (61.1)	62 (46.3)	
AJCC stage I	–	30 (16.0)	4 (7.4)	26 (19.4)	
*Grading, n (%)*					
G1	6	5 (2.7)	4 (7.5)	1 (0.8)	0.031
G2	–	108 (59.3)	32 (60.4)	76 (58.9)	
G3	–	69 (37.9)	17 (32.1)	52 (40.3)	
*HPV, n (%)*					
Negative	139	3 (6.1)	3 (21.4)	–	0.020
Positive	–	46 (93.9)	11 (78.6)	35 (100.0)	
*HIV, n (%)*					
Negative	0	177 (94.1)	43 (79.6)	134 (100.0)	< 0.001
Positive	–	11 (5.9)	11 (20.4)	–	
*MRI, n (%)*					
Yes	0	107 (56.9)	31 (57.4)	76 (56.7)	>0.9
No	–	81 (43.1)	23 (42.6)	58 (43.3)	
*PET/CT, n (%)*					
Yes	0	137 (72.9)	38 (70.4)	99 (73.9)	0.862
No	–	51 (27.1)	16 (29.6)	35 (26.1)	
*Radiation technique, n (%)*					
3D-CRT	0	90 (47.9)	26 (48.1)	64 (47.8)	>0.9
IMRT	–	98 (52.1)	28 (51.9)	70 (52.2)	
*Applied chemotherapy, n (%)*					
≥ 80% of planned dose	0	129 (68.6)	38 (70.4)	91 (67.9)	0.435
< 80% of planned dose	–	47 (25.0)	11 (20.4)	36 (26.9)	
No chemotherapy	–	12 (6.4)	5 (9.2)	7 (5.2)	

*3D-CRT* three-dimensional conformal radiotherapy, *IMRT* intensity-modulated radiotherapy

### Treatment

For staging purposes, 137 and 107 patients underwent PET-CT and MRI, respectively; 94 patients received both PET/CT and MRI. In total, 90 patients between 2004 and 2013 were treated with 3D-CRT, and 98 patients between 2010 and 2020 underwent IMRT. The median dose to elective lymph nodes was 45 Gy in both groups (3D-CRT: 41.4–50.4 Gy; IMRT: 37.8–51 Gy). Either a sequential or simultaneously integrated boost was delivered to the affected lymph nodes, with a median dose of 55 Gy (46.2 Gy–60 Gy) in the IMRT group and 59.4 Gy (51 Gy–60.4 Gy) in the 3D-CRT group, and also to the primary tumor, with a median dose of 55 Gy (46.2 Gy–64 Gy) in the IMRT group and 59.4 Gy (54 Gy–64.8 Gy) in the 3D-CRT group. Out of 188 patients, the initially planned radiotherapy was completed in 186 without treatment gaps of more than 4 days. One patient discontinued chemoradiotherapy at 46.2 Gy due to Fournier’s gangrene, while another patient’s radiotherapy was terminated one fraction before the planned end due to sepsis resulting in fatality.

Overall, 176 patients received at least one cycle of chemotherapy, with 166 patients receiving MMC/5-FU or MMC/capecitabine, 6 patients receiving MMC monotherapy, and 4 patients receiving other chemotherapy regimens. Among them, 129/176 patients were able to receive ≥ 80% of the planned dose, while a dose reduction to < 80% was necessary in 47 patients due to hematological toxicity or deteriorating general condition.

### Oncological endpoints

**Fig. 1:**
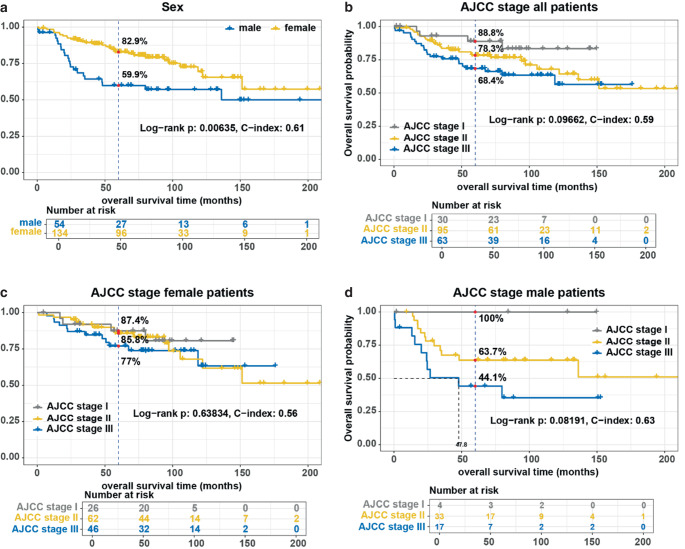
Kaplan–Meier analysis of overall survival for sex and American Joint Committee on Cancer (*AJCC*) stage. Censored patients are indicated by vertical ticks. Grey vertical dashed lines crossing the Kaplan–Meier curves mark (red dot) the 5‑year survival for every stratum. Given is the log-rank test *p*-value and the concordance index (*C‑index*) for every univariate Cox proportional hazard model. The analyses for **a** sex (male/female), **b** AJCC stage all patients, **c** AJCC stage female patients, and **d** AJCC stage male patients are shown

The 5‑year OS rate for the entire cohort was 76.5%; the 5‑year rates for DFS, FFR, and CFS were 76.5%, 86.6%, and 72.4%, respectively. The Kaplan-Meier analysis of OS by sex and AJCC stage is shown in Fig. 1, while the analyses of DFS, FFR, and CFS by sex and AJCC stage are additionally presented in the supplementary material.

In the univariate analysis, no significant associations were found for the radiotherapy technique used (3D-CRT vs. IMRT), N stage, or the use of MRI or PET/CT for staging. Female patients and patients younger than 66 years had significantly better OS. There was a trend towards better OS for patients with earlier tumors. A similar pattern was observed for DFS. There was a significant difference between sexes, indicated by an HR of 0.50 for females. Younger age and earlier tumors both showed a trend towards better DFS. In contrast to OS and DFS, no significant differences were found in FFR when comparing sex or age. However, a significantly better FFR was observed for earlier tumor stages. The CFS, like the OS and DFS, was significantly worse for male patients and patients with more advanced tumor stages. Older age showed a trend towards worse CFS (Table 2).

**Table 2:** Univarite analysis of prognostic and predictive factors for overall survival (OS), disease-free survival (DFS), freedom from recurrence (FFR), and colostomy-free survival (CFS) for the entire cohort and separately by sex

		OS		DFS		FFR		CFS	
		HR (95% CI)	*p*-value	HR (95% CI)	*p*-value	HR (95% CI)	*p*-value	HR (95% CI)	*p*-value
**Entire cohort**	Age (< 65 years)	0.57 (0.33–0.99)	0.04	0.62 (0.36–1.06)	0.08	1.05 (0.48–2.29)	0.90	0.61 (0.36–1.03)	0.06
	Sex (female)	0.47 (0.27–0.82)	0.01	0.50 (0.29–0.86)	0.01	0.52 (0.24–1.13)	0.09	0.56 (0.33–0.96)	0.03
	AJCC I	0.34 (0.12–0.99)	0.10	0.32 (0.11–0.93)	0.07	0.25 (0.06–1.10)	0.01	0.27 (0.09–0.78)	0.02
	AJCC II	0.70 (0.39–1.23)	–	0.68 (0.39–1.19)	–	0.36 (0.16–0.82)	–	0.61 (0.36–1.05)	–
	MRI	0.88 (0.50–1.53)	0.64	1.00 (0.57–1.73)	0.99	0.93 (0.42–2.04)	0.85	0.71 (0.42–1.21)	0.20
	PET/CT	0.97 (0.53–1.74)	0.91	1.00 (0.56–1.79)	0.99	0.58 (0.26–1.28)	0.17	0.91 (0.52–1.61)	0.75
	Radiation technique (IMRT)	0.86 (0.48–1.55)	0.62	0.85 (0.48–1.53)	0.60	0.99 (0.44–2.26)	0.99	0.74 (0.43–1.28)	0.27
	N1a	1.54 (0.85–2.78)	0.12	1.45 (0.81–2.60)	0.17	1.29 (0.55–3.05)	0.26	1.36 (0.77–2.4)	0.15
	N1b	0 (0-Inf)	–	0 (0-Inf)	–	0 (0-Inf)	–	0 (0-Inf)	–
	N1c	2.71 (1.05–7.01)	–	2.56 (0.99–6.6)	–	3.28 (0.95–11.38)		2.70 (1.05–6.92)	–
**Female patients**	Age (< 65 years)	0.56 (0.27–1.17)	0.12	0.61 (0.30–1.24)	0.17	0.72 (0.26–2.00)	0.53	0.63 (0.32–1.23)	0.17
	AJCC I	0.59 (0.19–1.84)	0.64	0.55 (0.18–1.68)	0.52	0.40 (0.09–1.87)	0.11	0.42 (0.14–1.27)	0.25
	AJCC II	0.80 (0.37–1.74)	–	0.75 (0.35–1.60)	–	0.33 (0.10–1.07)	–	0.70 (0.34–1.41)	–
	MRI	0.63 (0.30–1.34)	0.23	0.71 (0.34–1.47)	0.35	0.59 (0.21–1.70)	0.33	0.48 (0.24–0.96)	0.03
	PET/CT	0.80 (0.37–1.71)	0.56	0.88 (0.41–1.88)	0.74	0.74 (0.25–2.15)	0.57	0.75 (0.37–1.51)	0.42
	Radiation technique (IMRT)	0.51 (0.22–1.18)	0.11	0.50 (0.22–1.17)	0.11	0.66 (0.21–2.04)	0.47	0.45 (0.21–0.95)	0.03
	N1a	1.37 (0.61–3.05)	0.25	1.31 (0.59–2.91)	0.26	1.53 (0.51–4.61)	0.51	1.16 (0.55–2.44)	0.55
	N1b	0 (0-Inf)	–	0 (0-Inf)	–	0 (0-Inf)	–	0 (0-Inf)	–
	N1c	3.85 (0.88–16.79)		3.80 (0.87–16.54)		4.01 (0.50–32.49)		2.58 (0.60–11.02)	
**Male patients**	Age (< 65 years)	0.50 (0.22–1.17)	0.10	0.54 (0.24–1.22)	0.13	1.50 (0.40–5.69)	0.55	0.54 (0.23–1.26)	0.15
	AJCC I	0 (0-Inf)	0.08	0 (0-Inf)	0.10	0 (0 to Inf)	0.09	0 (0-Inf)	0.04
	AJCC II	0.50 (0.21–1.15)	–	0.53 (0.23–1.22)	–	0.34 (0.10–1.10)	–	0.43 (0.18–0.99)	–
	MRI	1.35 (0.56–3.21)	0.50	1.55 (0.65–3.67)	0.32	1.67 (0.48–5.86)	0.42	1.36 (0.57–3.24)	0.48
	PET/CT	1.46 (0.57–3.77)	0.43	1.34 (0.54–3.3)	0.53	0.48 (0.14–1.59)	0.22	1.44 (0.56–3.71)	0.45
	Radiation technique (IMRT)	1.86 (0.79–4.38)	0.15	1.79 (0.77–4.18)	0.17	1.93 (0.57–6.56)	0.29	1.69 (0.72–3.97)	0.22
	N1a	1.73 (0.7–4.26)	0.43	1.56 (0.65–3.77)	0.56	0.96 (0.24–3.85)	0.69	1.64 (0.66–4.03)	0.26
	N1b	NA	–	NA	–	NA	–	NA	–
	N1c	1.74 (0.48–6.33)	–	1.57 (0.44–5.64)	–	1.91 (0.39–9.49)	–	2.63 (0.72–9.62)	–

*AJCC *American Joint Committee on Cancer, *AJCC I* AJCC stage I, *AJCC II* AJCC stage IIA and IIB, *IMRT *intensity-modulated radiation therapy

The same univariate analysis was conducted separately for male and female patients. For male patients, there was a clear trend towards better OS, DFS, and FFR in earlier tumors as well as significantly better CFS. In contrast, in the female subgroup, there were no significant differences in OS, DFS, and CFS with respect to tumor stage. There was only a trend towards better FFR in earlier tumor stages (Table 2).

**Fig. 2:**
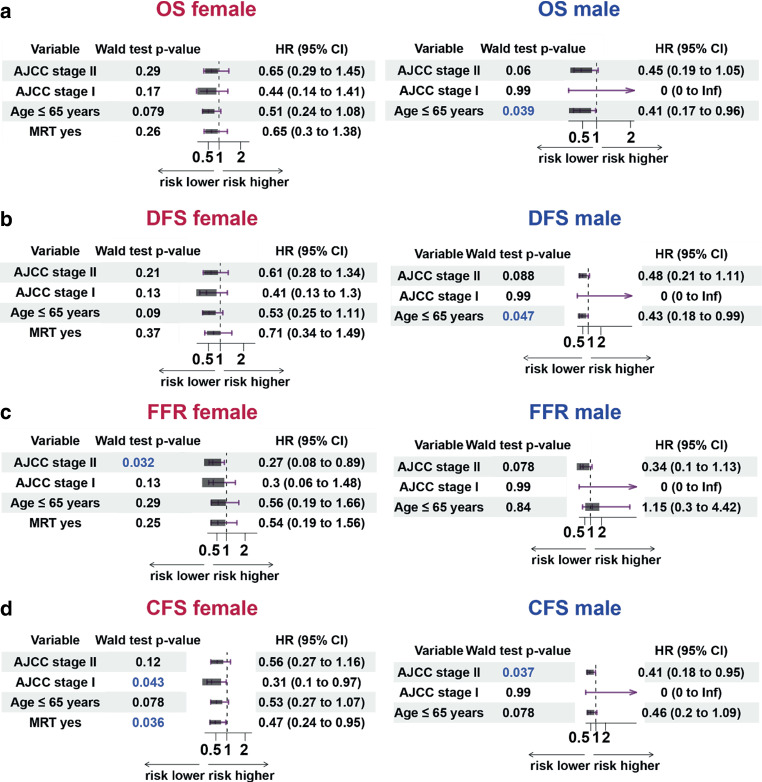
Forest plots of overall survival (*OS*; **a**), disease-free survival (*DFS*; **b**), freedom from recurrence (*FFR*; **c**), and colostomy-free survival (*CFS*; **d**) separated by sex. Given are the hazard ratios (*HR*), their 95% confidence intervals (*95%* *CI*) and the Wald test *p*-values

In the multivariate analysis of the entire cohort, male sex, older age (> 65 years), and advanced tumor stage were identified as significant risk factors for poorer OS, DFS, and CFS. Hazard ratios and confidence intervals are displayed in Table 3. In the subgroup analysis of female patients, neither age nor tumor stage were significant prognostic factors for OS, DFS, or CFS. However, within the male subgroup, age remained a significant prognostic factor for both OS and DFS (Fig. 2).

**Table 3:** Multivariate analysis of prognostic and predictive factors for overall survival (OS), disease-free survival (DFS), freedom from recurrence (FFR), and colostomy-free survival (CFS) for the entire cohort

	OS		DFS		FFR		CFS	
	HR (95% CI)	*p*-value	HR (95% CI)	*p*-value	HR (95% CI)	*p*-value	HR (95% CI)	*p*-value
Age (< 65 years)	0.44 (0.25–0.78)	0.01	0.47 (0.27–0.82)	0.01	0.76 (0.34–1.71)	0.50	0.48 (0.28–0.82)	0.01
Sex (female)	0.44 (0.25–0.77)	0.01	0.46 (0.27–0.80)	0.01	0.47 (0.21–1.03)	0.06	0.55 (0.32–0.94)	0.03
AJCC I	0.26 (0.09–0.77)	0.01	0.25 (0.08–0.74)	0.01	0.23 (0.05–1.05)	0.06	0.21 (0.07–0.61)	0.01
AJCC II	0.54 (0.30–0.98)	0.04	0.54 (0.31–0.97)	0.04	0.30 (0.13–0.70)	0.01	0.48 (0.28–0.84)	0.01

*AJCC *American Joint Committee on Cancer, *AJCC I* AJCC stage I, *AJCC II* AJCC stage IIA and IIB

## Discussion

In the current study, we report real-world data from a large monocentric cohort of 188 patients with a median follow-up of 83 months, all treated with (chemo)radiotherapy for anal carcinoma. Special focus was placed on sex-specific differences. Additionally, the prognostic significance of the recently updated AJCC staging system (now in its 9th edition) was tested.

Over 70% of our patients were female. The 5‑year OS rate in our study was significantly better for women at 82.9% compared to men at 59.9%. Similarly, male sex was identified as a significant risk factor for both CFS and DFS. Regarding FFR, a trend toward poorer FFR outcomes for male patients was observed, with a *p*-value of 0.09. Given the small number of events, we assume that a larger cohort might have demonstrated a statistically significant difference. Overall, these findings are in line with previously published data [13–18]. Arora et al. reported a median OS of 148 months for white women, 146 months for black women, 111 months for white men, and 82 months for black men [14]. Similarly, Koerber et al. demonstrated a 2-year OS of 63.5% for male and 89.5% for female patients in a cohort also treated in Germany [18]. Significant sex differences in terms of incidence and oncological endpoints have also been reported for a variety of other solid tumors. For example, Cook et al. showed high male-to-female incidence rate ratios (IRR) for numerous oncological diseases, with an IRR of 0.81 for anal carcinoma, indicating a higher incidence in women [2]. In another study, they also demonstrated elevated male-to-female mortality rate ratios (MMR) for a variety of tumors, with the most pronounced differences observed in lip cancer (MMR 5.5), hypopharyngeal cancer (MMR 4.5), esophageal cancer (MMR 4.1), and bladder cancer (MMR 3.4) [19]. Nakamura et al. demonstrated in a large meta-analysis of over 86,000 patients that female patients with non-small cell lung cancer had significantly better OS compared to male patients, regardless of tumor stage, histology, or smoking status [21]. Additionally, significant sex differences in survival favoring women were observed for oropharyngeal carcinomas, malignant melanomas, and colorectal carcinomas, among others [20, 23, 24]. The differences described in the literature highlight the need for sex- and gender-sensitive medicine. As early as 2015, the National Institutes of Health (NIH) called for the consideration of sex in the design and analysis of all studies [25]. Gender medicine encompasses all facets of sex-specific differences, considering not only biological distinctions but also varying gender identities and roles. The observed differences in incidence and oncological outcomes between men and women have been partially attributed to gender-specific variations in the use of healthcare services, such as screening programs. In our cohort, the proportion of female patients in AJCC stage I was significantly higher at 19.4% compared to 7.4% for male patients, suggesting that women may seek medical attention earlier.

Additionally, there is an evident difference in sex chromosomes, even in non-sex-related cancers, as well as in the varying levels of sex hormones [22]. Thus, the interplay between sex chromosomes and hormones impacts both the local drivers of carcinogenesis, including cancer-initiating cells and the components of the tumor microenvironment, as well as systemic factors such as cellular metabolism and the immune system [26]. Furthermore, significant sex-specific differences in the pharmacokinetics of many antitumor drugs have been documented. For instance, Mueller et al. reported a 26% higher elimination rate of 5‑FU in men compared to women [27]. Moreover, there are significant differences in body composition between sexes, with men having a substantially higher percentage of metabolically active fat-free body mass compared to women of the same height and weight [28]. Since calculation of the individual 5‑FU dose is based on body surface area, there are significant sex-specific differences in the circulating concentration profile. This impacts both the efficacy and toxicity of the treatment.

In addition to the sex-specific differences in oncological outcomes, our study also revealed a distinct difference in the prognostic value of the AJCC staging system within our cohort. For male patients, there was a strong trend towards better OS, DFS, and FFR, along with significantly better CFS for tumors in earlier stages. In contrast, the categorized AJCC stage (early/intermediate/advanced tumors) did not serve as a relevant prognostic factor in female patients.

As previously mentioned, the TNM and AJCC staging systems are intended, among other things, to function as prognostic tools to predict oncological outcomes. However, as stated by Janczewski et al., there was a lack of hierarchical prognostic order in the 8th version of the AJCC staging system, with 5‑year survival rates of 84.4%, 77.4%, 63.7%, 73.0%, 58.4%, 59.9%, and 22.5% for stages I, IIA, IIB, IIIA, IIIB, IIIC, and IV, respectively [11]. In the revised and recently published 9th edition, tumor stages IIB, IIIA, and IIIC have been updated, assigning greater importance to the T stage. Application of the 9th edition now shows a good hierarchical order, with 5‑year OS rates of 84.4%, 77.4%, 73.0%, 62.1%, 58.4%, 56.9%, and 22.5% for stages I, IIA, IIB, IIIA, IIIB, IIIC, and IV, respectively [11]. In the study by Janczewski et al., as well as in our cohort, women constitute the majority of patients, with 64.8% and 71.3%, respectively. Regarding ethnicity, 79% of the patients were non-Hispanic whites. The stage distribution patterns within the cohorts are similar, although we excluded stage IV patients from our analysis. A significant difference between the cohorts lies in the period during which patients were included in the analysis. Janczewski et al. included patients diagnosed between 2012 and 2017, while our cohort includes patients treated at our clinic between 2004 and 2020. Although the imaging modalities used for staging are not specified, the different inclusion periods suggest that a higher proportion of patients may have undergone MRI and/or PET/CT for staging. Given the known superior sensitivity and specificity of MRI and PET/CT compared to conventional CT alone, a stage shift may have occurred due to lymph node metastases not detected by CT alone. However, this factor alone does not explain the differing prognostic value of the AJCC stage for men and women in our cohort.

The main limitations of our study are the relatively small sample size and its retrospective character. There is evidence, for example from Grabenbauer et al., suggesting that tumors originating from the perianal skin are clearly associated with a worse prognosis [29]. Due to the retrospective nature of our analysis, a precise distinction between anal canal and anal margin carcinomas was not possible. Consequently, we cannot make any statements regarding the distribution of anal canal and anal margin carcinomas between men and women in our cohort or assess whether this had an influence on the oncological endpoints.

Notably, the limited number of male patients in the low-risk group, coupled with the absence of events in this group, affects the statistical significance of our findings. However, we anticipate that with a larger sample size or more events in this group, our analysis would reach statistical significance with respect to tumor stages. To address why the AJCC staging performed poorly in female patients in our cohort, we can only propose potential explanations at this stage. A possible reason might be the excellent 5‑year overall survival rate of nearly 83% across all stages for female patients. Such a high survival rate might render stage-based prognostic subdivision more challenging or potentially require a significantly larger cohort than ours to achieve statistical significance. As described, the retrospective analysis was conducted over an extended period. Consequently, there were variations beyond the investigated radiation techniques (3D-CRT vs. IMRT), which showed no significant difference. Notably, there were additional variations, particularly in target delineation and dose prescription, that were not specifically analyzed in the present study but which could potentially influence oncological outcomes.

Yang et al. developed a prognostic nomogram to predict 1‑, 3‑, and 5‑year survival incorporating the AJCC stage, sex, age, and whether or not radiotherapy was performed [30]. The nomogram, with a C-index of 0.684 and 0.730 in the training and validation groups, respectively, outperformed the AJCC stage (with C‑indices of 0.610 and 0.659), supporting the hypothesis that sex should be considered in prognostic assessments.

## Conclusion

The study highlights significant sex differences in terms of ASCC prognosis, with females showing better survival outcomes. The AJCC 9th edition staging system’s prognostic value differs between sexes, suggesting that sex should be considered in future prognostic assessments and treatment strategies for ASCC. The inclusion of sex as a prognostic factor in staging systems is supported.

## Supplementary Information

Kaplan-Meier analyses of DFS, FFR, and CFS by sex and AJCC stage; CONSORT diagram
