# Supplementary material for: Radio(chemo)therapy in anal cancer: evaluation of sex-specific disparities across AJCC stages
Source: Strahlenther Onkol. 2025 Feb 7;202(6):653–62. doi: 10.1007/s00066-025-02368-1 (PMC13216141; doi:10.1007/s00066-025-02368-1)
Supplement: Supplementary file 1 — Kaplan-Meier analyses of DFS, FFR, and CFS by sex and AJCC stage; CONSORT diagram [file 66_2025_2368_MOESM1_ESM.docx]

Supplementary Information

Radio(chemo)therapy in Anal Cancer: Evaluation of Sex-specific disparities across AJCC Stages

F. Fuchs^1^, P. Rogowski^1^, M. Rottler^1^, M. Shouman^1^, K. Heinrich^2,3^, F. Kühn^4^, C. Belka^1,3,5^, K. Unger^1,3,5^, F. Walter^1,3,5^

Frederik Fuchs
Department of Radiation Oncology, LMU University Hospital, LMU Munich, Munich, Germany
E-mail: [frederik.fuchs@med.uni-meunchen.de](mailto:frederik.fuchs@med.uni-meunchen.de)

SI Fig.1 Kaplan-Meier analysis of disease-free survival (DFS), freedom from recurrence (FFR) and colostomy-free survival (CFS) for sex and AJCC stage. Censored patients are indicated by vertical ticks. Grey vertical dashed lines crossing the Kaplan-Meier curves mark (red dot) the five-year survival for every stratum. Given is the Log-rank test p-value and the concordance index (C-index) for every univariate Cox-proportional hazard model. The analyses for **A** DFS, **B** FFR, **C** CFS are shown.

SI Fig.2 CONSORT diagram (RT: Radiotherapy, VIN: Vulvar Intraepithelial Neoplasia)


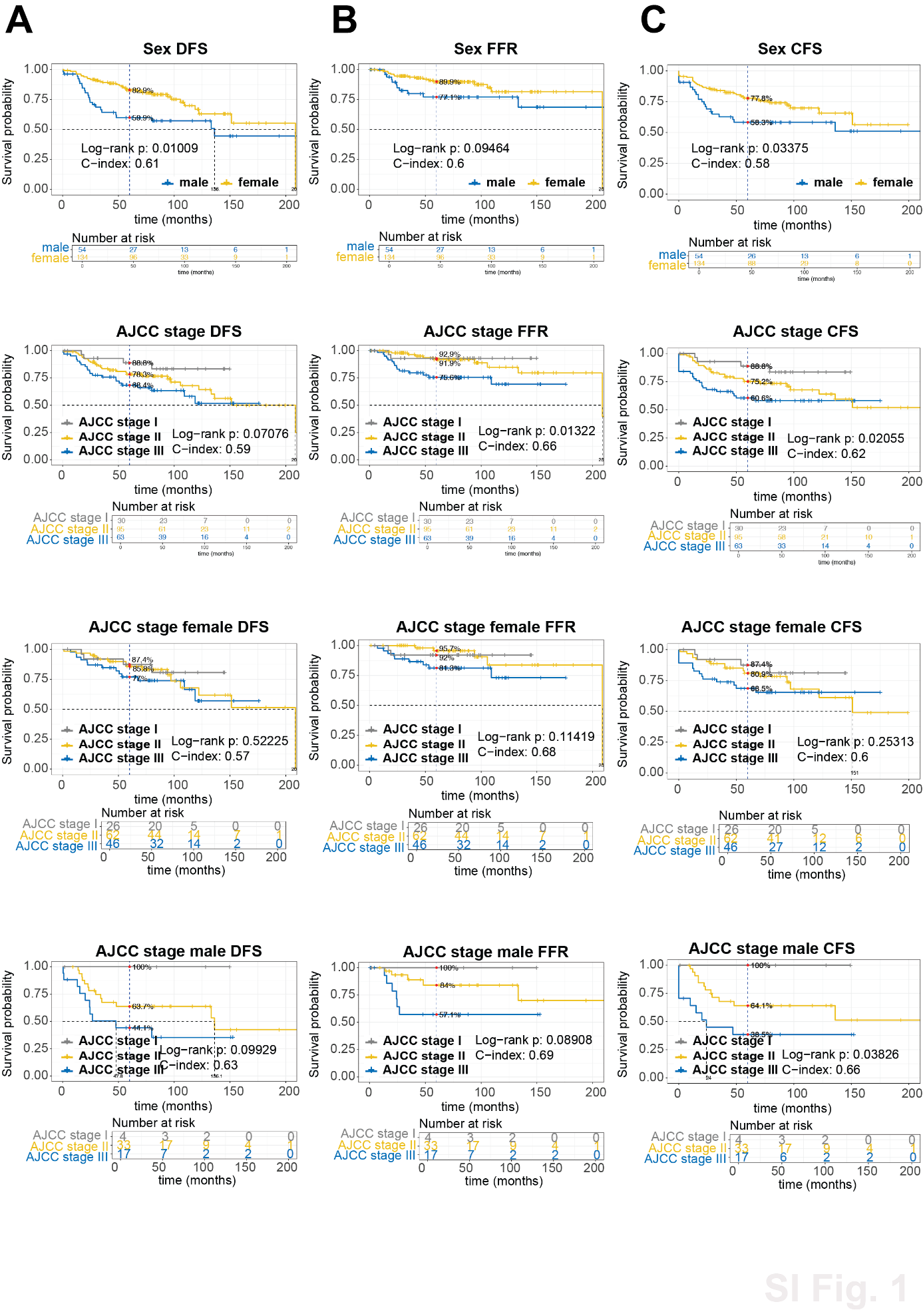


**SI Fig. 1** Kaplan-Meier analysis of DFS, FFR and CFS for sex and AJCC stage


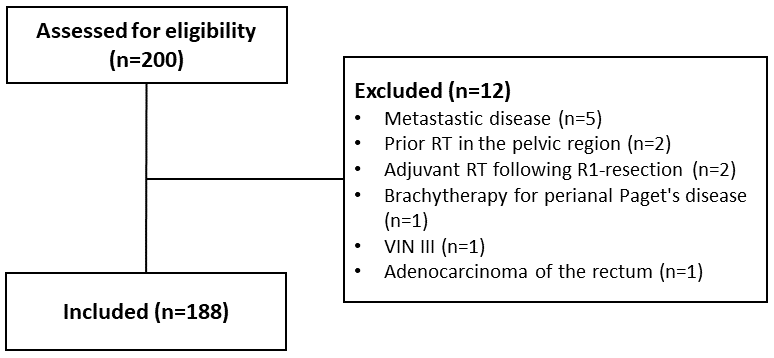


**SI Fig. 2** CONSORT diagram (RT: Radiotherapy, VIN: Vulvar Intraepithelial Neoplasia)
